# Effect of Packaging and Portioning on the Dynamics of Water–Fat Serum Release from Fresh Pasta Filata Soft Cheese

**DOI:** 10.3390/foods11030296

**Published:** 2022-01-22

**Authors:** Jakub Biegalski, Dorota Cais-Sokolińska, Jolanta Wawrzyniak

**Affiliations:** Department of Dairy and Process Engineering, Faculty of Food Science and Nutrition, Poznań University of Life Sciences, ul. Wojska Polskiego 31, 60-624 Poznan, Poland; jakub.biegalski@up.poznan.pl (J.B.); jolanta.wawrzyniak@up.poznan.pl (J.W.)

**Keywords:** water–fat serum, artificial neural network model, cheese, portioning, packaging

## Abstract

The aim of the present study was to analyze the impact of cheese fragmentation and packaging on the dynamics of water–fat serum released from pasta filata cheese made from cow’s milk and its mixture with sheep’s milk. The addition of sheep’s milk reduced the amount of leachate from the vacuum-packed cheeses and did not cause as much loss of gloss as in the case of cow’s milk cheeses. This was also reflected in the microscopic images of the cheese samples. Consumers showed less acceptance of cow’s milk pasta filata cheeses than cheeses made with a mixture of cow’s and sheep’s milk (they had the same fat content, acidity, hardness, and oiling-off, but better stretching). The data describing water–fat serum release from pasta filata cheese within 24 h of unpacking was modeled with the use of the feed-forward artificial neural networks, whose architecture is based on Multi-Layer Perceptron with a single hidden layer. The model inputs comprised four independent variables, including one quantitative (i.e., time) and the other qualitative ones, which had the following states: type of raw material (cow’s milk, cow-sheep’s milk), way of sample portioning (whole, quarters, slices), packing method (vacuum packed and packed in brine).

## 1. Introduction

Pasta filata cheese production technology is based on the processing of milk from various species of mammals. Traditionally, cow’s milk is used to produce Provolone del Monaco cheese [1,2], buffalo’s milk produces Mozzarella di Bufala Campana PDO [3,4,5], and sheep’s milk is used to produce Oscypek PDO cheese [6], semi-hard Kasseri PDO cheese [7,8] and Vastedda Della Valle del Belice PDO soft cheese [9,10]. Among these cheeses, Kashar cheese, which traditionally originates from Turkey, can be made from cow’s milk, sheep’s milk, or a mixture of both [11,12]. Currently, the adaptation of the production technology of many pasta filata cheeses allows their more widespread production from cow’s milk and, therefore, more accessible raw material. This is the case for Mozzarella cheese or Caciocavallo cheese. However, as with any modification of the raw material and technical/technological parameters, it is associated with obtaining cheeses with properties different from the original [4,13].

Mozzarella is one of the most popular cheeses around the world. Its high or low moisture depends on fat content in the dry matter. For example, when content of fat in dry matter (*m/m*) is ≥30% but <40%, then the corresponding minimum dry matter content (*m/m*) for low- and high-moisture Mozzarella is 39% and 26%, respectively [14]. It is characterized by a fresh, milky taste and an exceptionally soft texture, and is stored in covering liquid. This type of cheese shows specific properties, where serum leachate after portioning of the cheese is probably the most particular [15,16]. The composition of the covering liquid may be different, including, among others, water, lactic acid or citric acid, NaCl, and CaCl_2_. It is a type of brine that maintains high moisture in the cheese, usually above 60%, as well as very soft texture, and prevents the formation of rind on the surface [16,17]. Nevertheless, the use of a covering liquid contributes to the reduction of the shelf-life due to high moisture content and water activity, and mass transfer between the cheese matrix and the serum phase [16,17,18]. The presence of water redistribution and enhanced water-holding capacity in cheese during longer storage has been described by Gonçalves et al. [19].

The physical state of the water in cheese critically influences both structural and functional properties. Cheese has a bi-continuous gel structure consisting of a porous protein matrix (casein) interrupted by fat [20]. Some of the water in cheese is located near or inside fat clusters [21], while most of the water is found in the porous casein matrix [20]. The water fractions of cheese are generally divided into a matrix associated with casein and unbound free serum water [20].

Mozzarella cheese is widespread among consumers, not only for its sensory properties, but also due to its nutritional and health benefits; however, one undesirable feature in the consumption or production and industrial use of Mozzarella is leachate that appears after its unpacking. This phenomenon is not only negatively perceived by consumers, but also (due to the reduction in cheese mass) is a source of losses for producers. Considering the impact of leachate on the profitability of production and the consumer impression of pasta filata cheese, it is necessary to identify the conditions that favor its formation. To the best of our knowledge, reports have not comprehensively examined the effect of raw material used to manufacture cheese and its post-production processing on the amount of leachate. Therefore, the aim of the present study was to analyze the impact of cheese fragmentation and packaging on the dynamics of water–fat serum release from pasta filata cheese made from cow’s milk and its mixture with sheep’s milk. To enable easy use of the obtained results in industrial practice, the artificial neural network model of water–fat serum released as a function of the above-mentioned parameters was elaborated. In addition to the scientific cognitive value, the developed model will be useful for technologists designing specific properties of cheese and the food industry, especially HoReCa (Hotel, Restaurant, Catering).

## 2. Materials and Methods

### 2.1. Cheese-Making Protocols

A detailed description of cow’s and sheep’s milk used and the procedure for making pasta filata cheese was described by Biegalski et al. [22]. Half of the produced cheeses were vacuum packed using a vacuum sealer (A300/16 type, Multivac GmbH & Co.KG, Wolfertschwenden, Germany). The other half were packed in brine and stored at 3 °C. PA/PE bags with a thickness of 0.08 mm were used. The produced cheeses were shaped into spheres (220 g, Ø = 7 cm). The research material was a whole sphere of cheese, quartered cheese, and sliced cheese.

### 2.2. Experimental Design

The cheese was quartered by slicing along the geometric center horizontally and vertically to obtain 4 equal quarters of cheese. Slicing was carried out with a food slicer (R506E, Gorenje d.d., Valenje, Slovenia) to obtain 1 cm thick slices. Table 1 shows the parametric data of fresh pasta filata cheese before and after portioning. The differences in dimensions were closely related to the way the ball of cheese was cut. Hence, the area of the cheese slice was greater than that of the quarter.

The various parameters of cheese quality, sensory test, and the amount of water–fat serum released from cheese were rated after production (after 2 days of storage in packaging at 3 ± 0.5 °C). Storage time for all samples was 2 days after production, which imitates the period of time from the end of production to the moment the product goes on sale. Test specimens were taken from different production batches (*n* = 6). The cheese was prepared in a pilot plant scale and each batch was analyzed twice.

### 2.3. Composition and Acidity of Cheese

The composition of the cheese was determined according to moisture [23], protein [24], and fat [25] content. Total protein was calculated as: (TN − NPN) × 6.38. pH was measured using a CP–402 pH-meter (Elmetron, Zabrze, Poland) equipped with a IONODE IJ44A electrode (Ionode Pty. Ltd., Tennyson, Australia). The titratable acidity values were expressed as Soxhlet–Henkel degree (SH, 1 SH = 0.0225 lactic acid %).

### 2.4. Profile Texture Analyses and Oiling-Off

The hardness and stretching of the cheeses were measured using a texturometer (Stable Micro Systems Ltd., Surrey, UK) with attachments: A/WEG—hardness (Pre-Test Speed 1.0 mm/s, Test Speed 2.0 mm/s, Post-Test Speed 10.0 mm/s, distance 10.0 mm); A/CE (stretch quality) attachment with a PT 100 temperature sensor (test speed 20.0 mm/s, post-test speed 20.0 mm/s, distance 270 mm, temp. 55 °C, samples of 60 g). Results were recorded using Texture Exponent E32 version 4.0.9.0 software (Godalming, Surrey, UK).

Oiling-off (fat-ring test) was determined according to the method of Schenkel et al. [26] and Hartmann et al. [27]. The free oil formation was expressed as the percentage of the area soaked by free oil relative to the area of the total filter paper.

### 2.5. Gloss Measurement and Microstructure

The gloss was measured using the DT 268 gloss meter (TestAn, Gdańsk, Poland), measurement geometry 60.

All samples were evaluated using optical microscopy. Observations were conducted on pieces taken from the central layers of the cheese mass. The fragment dimensions were as follows: width 2 mm, height 2 mm, and thickness 0.3 mm. Samples of cheeses were deposited onto a glass slide surface and covered with a cover slip for observation under an optical microscope ProteOne (Delta Optical, Mińsk Mazowiecki, Poland). Observations were made at 1000× magnification using a ProteOne semiplanachromatic objective (Delta Optical, Mińsk Mazowiecki, Poland) with oil immersion. Images were taken using DLT-Cam PRO microscope camera (Delta Optical, Mińsk Mazowiecki, Poland).

### 2.6. Acceptability of Appearance and Consumer Penalty Analysis

In the sensory test, consumers (*n* = 84; 50 female, 34 male; ages 29 to 69; M_age_ = 35.5, SD = 8.71) were asked to indicate how much they liked or disliked each product on a 9-point hedonic scale (9 = like extremely; 1 = dislike extremely) based on appearance. Each consumer was given 12 cheese samples for evaluation: whole sphere of cheeses, quartered cheeses and sliced cheeses, packed in brine and vacuum packed, made from cow’s milk and a mixture of cow’s and sheep’s milk. The samples were assessed between 5–6 h after removing from the packaging and portioning (according to preliminary observations, this is the average storage time of the cheese after unpacking by the consumer). Samples were held and served at 6 °C in a refrigerated display case (YG-05025, YATO, Wrocław, Poland). Any assessor who rated the sample at a level of 1 to 4 (dislike) had to rate shininess, leachate and compactness using a 5-point just-about-right (JAR) scale. For this purpose, the methodology described by Costa et al. [28] was used. Ratings consisted of 1 = not enough, 3 = ideal, 5 = too much.

### 2.7. Modeling Process

#### 2.7.1. Datasets

The model of water–fat serum release from pasta filata cheese was developed based on experimental data, which described the volume of liquid phase (mL) collected within 24 h after cheese unpacking (according to preliminary research, it is the maximum storage time of the cheese after unpacking by the consumer). The data included observations recorded from cheeses that differed in terms of packaging method, degree of sample fragmentation and type of raw material used in production. During modeling, data were randomly divided into three groups, training, testing and validation. The training dataset used for the network learning process consisted of 604 points, which accounted for 70% of all cases. The other two datasets, the set of testing data used to evaluate the network during its training and the set of validation data not involved in the construction of the model, was used for the final model verification, each contained 130 points, i.e., 15% of the full data set.

#### 2.7.2. Model Development

Artificial neural networks (ANNs) are a universal approximating system capable of mapping dependences existing in multidimensional datasets. ANNs do not require a priori knowledge of the relationship between process variables and offer a simple and straightforward approach to problem identification; hence, they constitute a highly promising modeling technique particularly in the case of non-linear phenomenon [29]. The most commonly used neural networks consist of several layers of neurons (input, one or more hidden and output layers). Determination of the number of hidden layers, the number of neurons in each of them, and the type of activation functions in neurons of the hidden and output layers is part of the neural network design process. The values of network parameters (weights, biases) are estimated in the optimization process to allow the network to best map the set of independent variables constituting the input signals into the set of dependent variables constituting the output signals.

In our study, feed-forward networks based on Multi-Layer Perceptron (MLP) with a single hidden layer were applied to develop the model of water–fat serum released from pasta filata cheese within 24 h of unpacking. The model inputs were comprised of four independent variables. One was quantitative, i.e., time, while the others were qualitative and took the following states, i.e., type of raw material (cow’s milk, cow-sheep’s milk), the method of sample portioning (whole, quarters, slices), and the packing method (vacuum packed and packed in brine). When designing the network, each of the variables were assigned to several neurons equal to the number of states. For a specific state of a given qualitative input variable, only one of the assigned neurons could take one of two values, i.e., 0 (inactive) or 1 (active). In turn, the dependent variable, which was the leachate volume, was taken as the output of the network. During model development, network topologies based on MLP containing a different number of neurons in the hidden layer (from 2 to 20), various types of activation function in neurons of hidden layer (i.e., hyperbolic tangent (Tanh), logistic (Log) and exponential (Exp)) and linear activation function (Lin) in the neurons of output were examined. The network parameters (weights, biases) were determined using the Broyden–Fletcher–Goldfarb–Shanno (BFGS) training algorithm. The total number of tested ANN topologies was 57,000 (3 types of activation functions in neurons of hidden layer × 19 sizes of hidden layers × 1000 repetitions for each structure). The performance quality of each tested network was assessed during the model-designing process based on the values of training and test errors. The generalization capability of examined networks was evaluated based on a validation error. All errors were calculated with the use of the sum-of-the-squares error function.

### 2.8. Statistical Analyses

Statistical analysis was carried out using TIBCO Statistica data analysis software, version 13.3.0 (TIBCO Software Inc., Palo Alto, CA, USA). Results are presented as mean ± standard deviation (SD) of triplicate of each analysis carried out in experiments performed in duplicate. A critical level of significance of α = 0.05 was used throughout the study. The influence of milk composition on chemical and physicochemical characteristics of pasta filata cheeses was evaluated by one-way analysis of variance (ANOVA). The effect of milk composition, the packaging method and the sample fragmentation on the leachate amount within 24 h of cheese unpacking was investigated using multivariate analysis of variance (ANOVA). The significance of the effect of individual factors and their interactions was assessed using the F test, while the significance of differences between the mean values of leachate volumes was determined using the post-hoc Tukey’s HSD test. Each analysis of variance began with the verification of its assumptions including examination of the probability distribution for measured variable and homoscedasticity in the standard deviation of its value using the W Shapiro–Wilk and Levene tests, respectively. The evaluation of the ANN model of water–fat serum released from fresh pasta filata cheese performance was carried out based on the determination coefficient (R^2^) and root mean square error (RMSE). 

## 3. Results

### 3.1. Composition and Physicochemical Properties of Fresh Pasta Filata Cheese

Cheese from cow’s and sheep’s milk in 70:30 proportion (CS), compared to cheese from only cow’s milk (C), contained less moisture by approx. 6% (*p* < 0.05, Table 2), whereas the protein content in CS cheese was approx. 17.2% higher than in C cheese (*p* < 0.05). Both groups of examined cheese samples had comparable fat content (*p* > 0.05). Additionally, no difference in hardness and oiling-off was observed. Moreover, the addition of sheep’s milk had no effect on the acidity and pH of the cheeses (*p* > 0.05). pH values were 5.15 and 5.14, respectively. These results were comparable to those reported by Alinovi et al. [16], whose cow’s milk Mozzarella cheese had pH value of 5.92 (*p* < 0.05). Significant differences were observed in the case of the stretching parameter that was greater for CS (*p* < 0.05). The string length of CS cheese was 131.6 mm, which was longer than that of C cheese by approx. 2.9%. Similar results were presented by Fife et al. [30], with string length of 125 mm.

Due to their complex structures, cheeses are an example of food with viscoelasticity [31]. According to Muliawan and Hatzikiriakos [32], Mozzarella is viscoelastoplastic at room temperature, but above 60 °C it is a viscoelastic material. It has been shown that the yield point of Mozzarella cheese gradually decreases with increasing temperature. Deformation, fracture, and friction during cutting are closely related to viscoelastic properties [33].

### 3.2. Surface Condition Assessment

**Figure 1:**
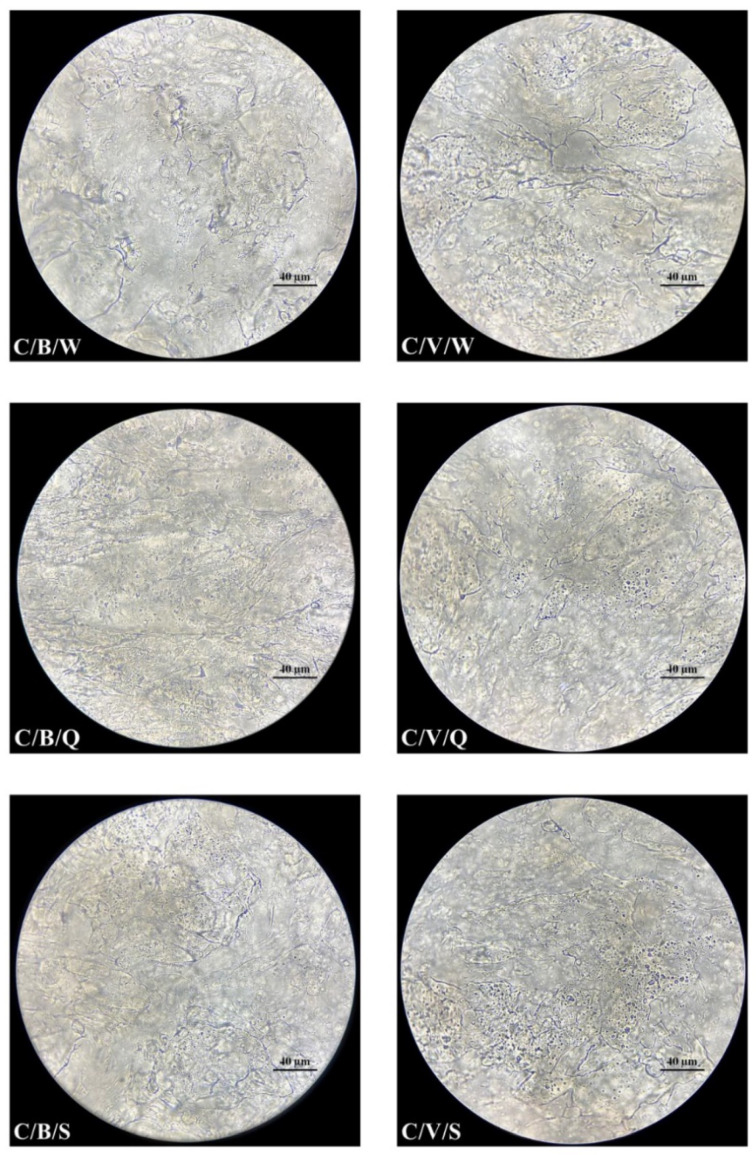
Microstructures of pasta filata cheeses from cow’s milk (optical microscopy, 1000× objective); Milk (used in cheese production): C: cow’s milk; Packaging (method): B: Packed with brine; V: Vacuum packed; Fragmentation (method): W: Whole sphere; Q: Quarters; S: Slices.

**Figure 2:**
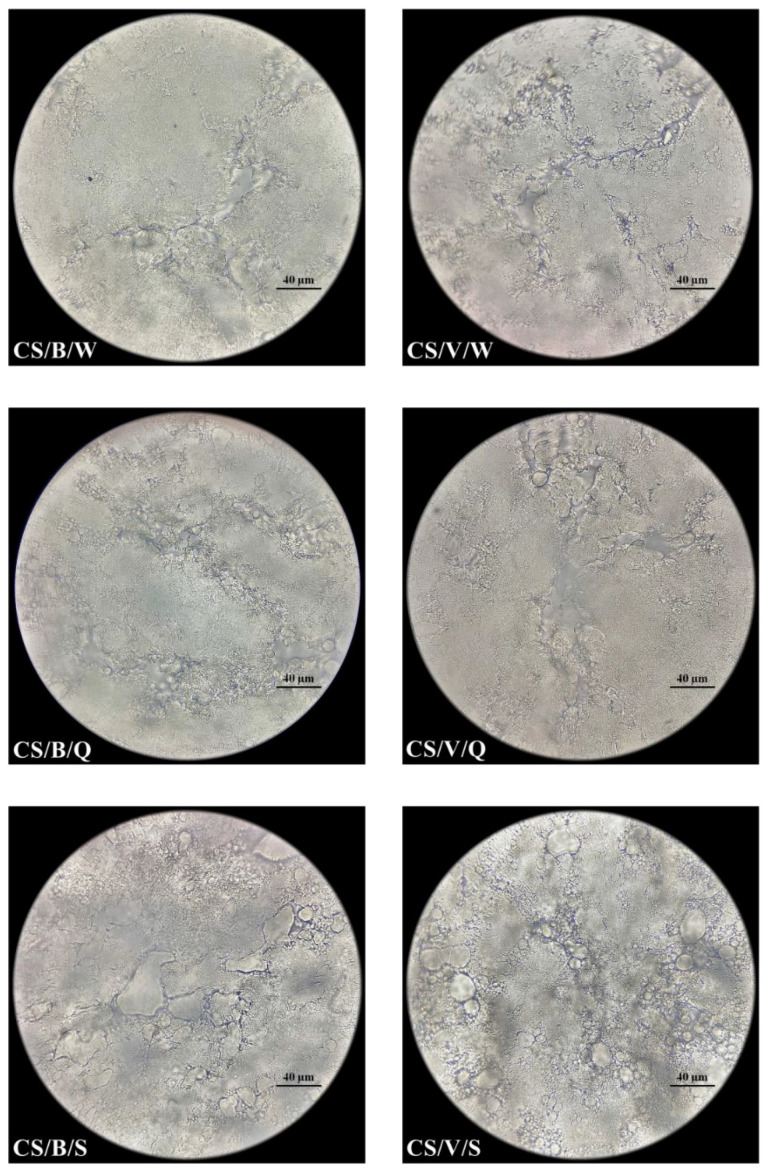
Microstructures of pasta filata cheeses from mixture of cow’s and sheep’s milk (optical microscopy, 1000× objective); Milk (used in cheese production): CS: cow’s and sheep’s milk in proportion 70:30; Packaging (method): B: Packed with brine; V: Vacuum packed; Fragmentation (method): W: Whole sphere; Q: Quarters; S: Slices.

Images of the samples observed with the microscope reflect the size of leachate (Figure 1 and Figure 2). The addition of sheep’s milk reduced the amount of leachate from the vacuum-packed cheeses. Thus, it did not lead to such a high loss of gloss as in cow’s milk cheeses. This is demonstrated by the plasma/serum retained in the channels of the cheese structure. The structure of vacuum-packed CS cheese is more compacted (packed/thickened) than that of C cheese packed in the same way. The cheeses packed in brine turned out to have higher gloss and higher plasma/serum content.

During cutting, shearing stresses cause structural damage and propagate its fracture. The applied load along with the higher speed increases the stiffness of the cut material. Thus, the deformation zone caused by the pressure of the blade is reduced. Vandenberghe et al. [34] proposed a critical stress and distance criterion for crack propagation in portioning models of cheese. As emphasized by Bremer and Matthiesen [35], there is a difference between portioning research and slicing and hence there is a need to conduct experimental research on slicing to define the ideal process.

**Figure 3:**
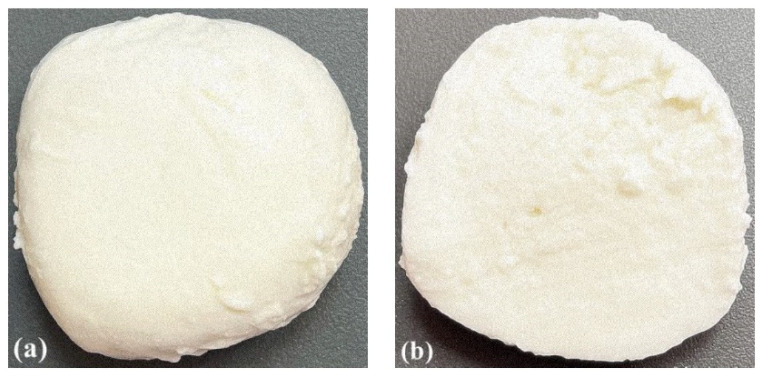
Outer layer (**a**) and inner layer (**b**) of pasta filata cheese from cow’s and sheep’s milk in proportion 70:30.

With cutting force, the appearance and roughness of the cut surface is connected [36]. This is an important criterion for food quality [37]. As per Chen [38], surface texture can be captured by the senses by visual, tactile handfeel, and tactile mouthfeel senses. It can be assessed using sensory panel tests or by physical instrument tests. In our research, cutting into quarters as compared to slicing was probably associated with less deformation and less friction. This could have had an impact on the structure of the obtained portion. However, the surface of the cheese portions after quartering and slicing, in our opinion, was visually just as shiny and rough, but significantly different from the outer surface (Figure 3). The gloss of the outer layer was 1.4 GU (sample C and CS, *p* > 0.05), and the internal one was 0.8 GU (sample C and CS, *p* > 0.05).

### 3.3. Dependence of Cheese Packing and Portioning on Acceptability of Appearance

Consumers indicated the highest acceptability at the “like extremely” level for CS cheese made from a mixture of cow’s and sheep’s milk in the proportion of 70:30 (CS) (Table 3). The highest values were observed for whole-sphere cheese samples packed with brine. In general, C pasta filata cheeses were less accepted by consumers than CS cheeses. This was observed especially for vacuum-packed C cheese served in the form of quarters and slices.

Dissatisfaction by consumers was observed in the case of vacuum-packed quarters and slices from C cheese, and for samples in the form of slices from CS cheese, also vacuum-packed (Table 4). The main reason for consumer dissatisfaction with C cheeses stemmed from not enough of shininess, for samples served in the form of quarters and slices. “Not enough shininess” response was also observed in the case of CS cheese, in the form of slices. Consumers largely demonstrated dissatisfaction due to excessive leachate from these cheeses. According to respondents, these cheeses also largely lacked compactness. The consumer penalty analysis of the just-about-right (Table 4) showed that the most “not enough shininess” responses was in the case of samples from cow’s vacuum-packed cheese, served in form of quarters (C/V/Q) (61.3% of respondents). Most “too much leachate” (97.7%) and at the same time “not enough compactness” (83.7%) responses was in the case of samples from cow’s vacuum-packed cheese, served in form of slices (C/V/S).

Cheese portioning is necessary during consumption, e.g., in salads, pizza topping, and sauces. For portioned cheese to be appealing to consumers, food processors and food-service industries, it must have a stable structure, with the least possible leachate of water and free oil [39]. The mobility of water within the cheese matrix is related to features strictly responsible for the functionality of the cheese, e.g., melting and texture. Cheeses in which the water moves more are considered to exhibit greater meltability and softness. When water is strongly bonded in the matrix, the cheese is brittle and non-melting [39]. The sensory descriptive analyzes of Mozzarella cheese during shelf-life reported by Cincotta et al. [40] showed that the structural features of the cheese surface are important regarding sensory acceptability. This research showed that features such as white color, smooth surface, firmness and juiciness are noticeable by consumers at an average level of 8.63, 6.74, 8.06, and 7.03, respectively (using a nine-point intensity scale, where 1 = not perceptible and 9 = strongly perceptible). This demonstrates that these characteristics are relevant to the consumer in the case of pasta filata cheeses. Additionally, juiciness is assessed integrally by the amount of the liquid fraction in the cheese mass. The leachate of this phase is unacceptable to the consumer. Therefore, every effort should be made to minimize the amount of leachate. Uzun et al. [41] examined buffalo milk Mozzarella cheese, and presented similar results to those reported by Cincotta et al. [40]. Juiciness, smoothness and tenderness are some of the highest rated descriptive attributes, and confirms the assumption that these features are important for consumers.

### 3.4. Effect of Process Parameters on Water–Fat Serum Release from Fresh Pasta Filata Cheese

**Figure 4:**
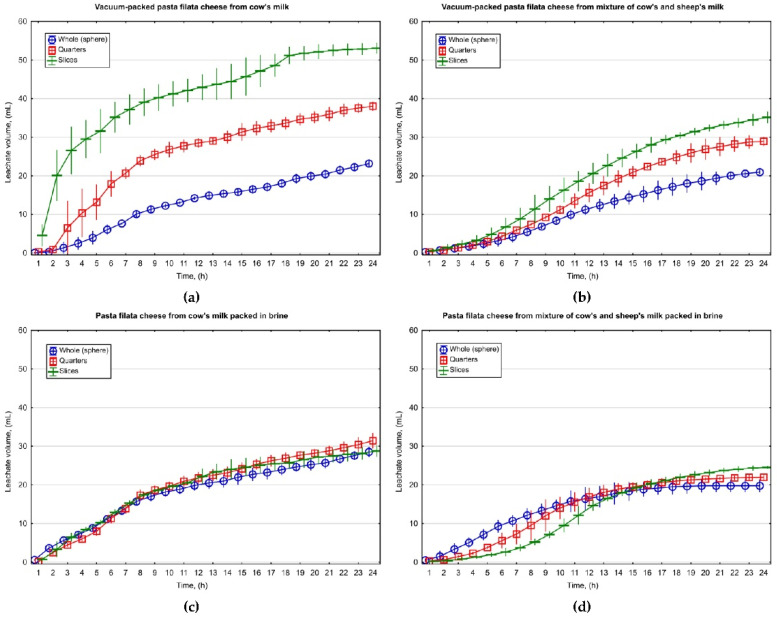
The amount of water–fat serum released from fresh pasta filata cheese made from cow’s milk (**a**,**c**) and its mixture with sheep’s milk (**b**,**d**) within 24 h after unpacking depending on the degree of sample fragmentation and packaging method.

**Figure 5:**
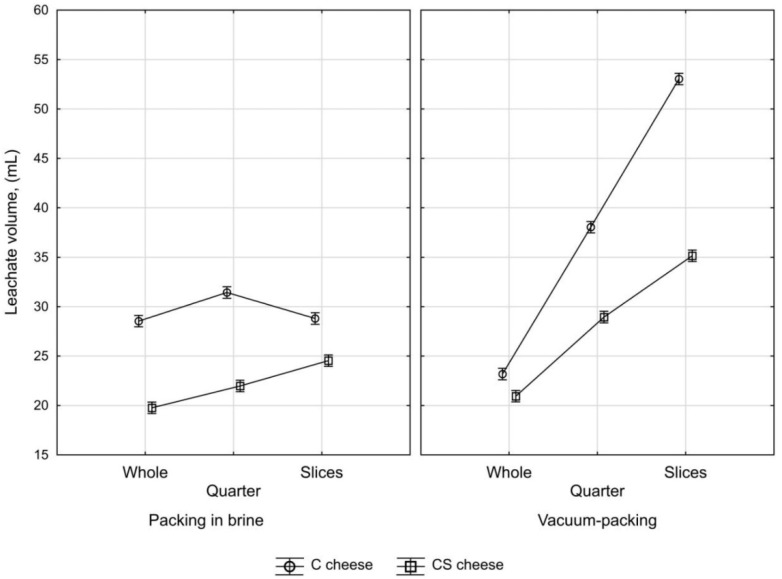
The effect of raw material type, the degree of sample fragmentation and packing method on the average amount of water–fat serum release from fresh pasta filata cheese made from cow’s milk (C cheese) and its mixture with sheep’s milk (CS cheese) within 24 h after its unpacking.

Figure 4 and Figure 5 show the amount of leachate flowing out of C and CS cheeses collected after their unpacking as a function of the degree of cheese fragmentation, packaging method and time. Data presented in the diagrams show that vacuum packing increased leachate amount. Cheese fragmentation, leading to an expansion of its surface (an increase in surface-area-to-volume ratio, as well as reduction of the average distance from the inside of the sample to its surface), significantly enhanced the vacuum-packing effect on the amount of water–fat serum release. The influence of vacuum packing on the leachate of liquid phase from cheese mass was higher for cheese made from cow’s milk than that from a mixture of cow’s and sheep’s milk. In the tested samples of vacuum-packed C cheese, the degree of extension of cheese fragmentation resulted in increased leachate volume and after 24 h it ranged from 23.18 ± 0.39 mL for spherical cheese samples to 53.04 ± 0.53 mL for sliced samples, whereas CS cheeses were in the range of 20.94 ± 0.18–35.15 ± 0.57 mL. Packing in brine diminished the effect of cheese portioning on the amount of leachate. The reduced amount of leachate in brine-packed cheese in comparison to vacuum-packed cheese may be due to lactose, of which brine is a rich source that promotes the formation of an orderly cross-linking of water in the channels of stretched casein fibers [22], which may increase water retention in the cheese mass.

The composition of milk used in the production of the examined cheeses affected the volume of water–fat serum release. The addition of sheep’s milk during cheese manufacturing contributed to increased water retention in cheese mass (Figure 4). According to previous studies, water retention in Mozzarella cheese may be related to the fat content in its mass [42]. The results of the three-factorial analysis of variance also confirmed that the composition of milk used in cheese production (MC), the degree of sample fragmentation (SF), as well as packing method (PM) and their interactions have a significant impact on the amount of leachate generated from cheese (Table 5). Post hoc analysis with the application of Tukey’s test revealed increased leachate volume associated with elevated cheese fragmentation, which was particularly important for vacuum-packed samples of cheese (Figure 5).

### 3.5. Model of Water–Fat Serum Release from Fresh Pasta Filata Cheese

Experimental data describing the leachate amount collected within 24 h of unpacking the pasta filata cheeses from C cheese and CS cheese with various degrees of sample fragmentation, vacuum-packed and packed in brine were used to develop the ANN model. In previous research, ANNs in the form of MLP with a single hidden layer were sufficient in describing a non-linear phenomenon occurring in food processing [43,44,45,46]. Therefore, in our study, such topologies were used to model the amount of leachate from pasta filata cheese. The number of input and output neurons is usually determined by the nature of the analyzed problem. In the presented research, the input signals of the designed neural network model were the raw material used for cheese manufacturing, the packaging method, the degree of cheese fragmentation and time, whereas the output of networks was volume of leachate. The number of neurons in the hidden layer was one of the elements of the neural network topology that was defined during the network optimization process. Since no universal method is available to determine the network topology, the number of hidden layers, the number of neurons in hidden layers, the type of activation functions in neurons of hidden and output layer, the issue is usually solved by a trial-and-error approach [29]. It is worth noting that too many neurons in the hidden layer led to the memorization of specific cases and the inability of the network to generalize data, while too few neurons leads to low-quality prediction. In the study, the network topologies with a single hidden layer containing between 2 and 20 neurons equipped with activation functions in the form of Tanh, Log, Exp and one neuron with Lin activation function in the output layer were examined. The obtained results revealed that the group of networks with 11–13 hidden neurons containing logistic activation function provided the best prediction of the leachate volume. The topology of those networks provides a balance between the simplicity of construction and quality of predictions. In this group of ANNs, MLP 4(8)-11-1 was the best one and was taken as the neural network model of water–fat serum release from fresh pasta filata cheese. The errors computed for the selected ANN-MLP network during network designing process are presented in Table 6.

**Figure 6:**
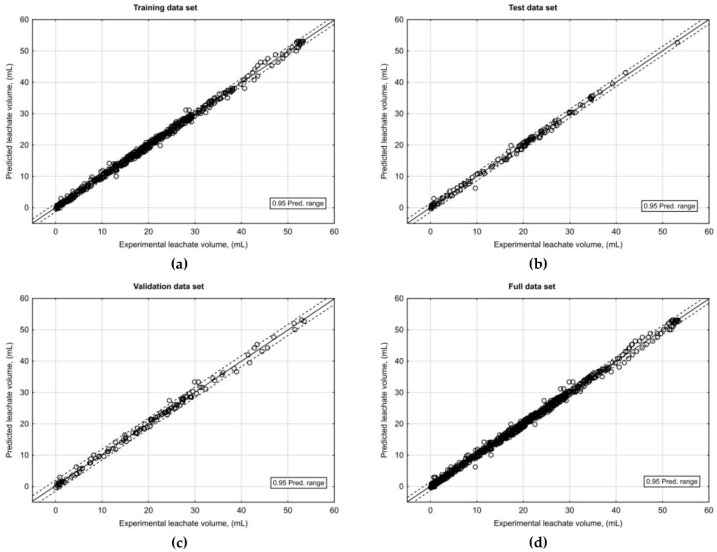
Predictability of ANN-MLP model of water–fat serum release from pasta filata cheese. The solid line represents a perfect fit between experimental and predicted values for (**a**) training, (**b**) test, (**c**) validation and (**d**) full datasets.

The results of the prediction accuracy assessment of the developed ANN-MLP model of water–fat serum released from pasta filata cheese are summarized in Table 7. The external validation of the developed ANN model based on experimental results that was not used in model development (validation dataset) showed that the selected network was characterized by high goodness of fit to experimental data (R^2^ = 0.996) and low RMSE value (0.765). The predicted amounts of water–fat serum released from pasta filata cheese plotted against the observed values (Figure 6) showed a high linear correlation between the experimental and predicted data that was confirmed by high correlation coefficient (R = 0.998).

## 4. Conclusions

In the present work, we investigated the effect of the portioning of cheese into quarters and slices of previously vacuum-packed or packed-in-brine cheese on the leachate of the water–fat serum. The results showed that the amount of leachate affected by the portioning of cheese was negatively perceived by consumers. Dissatisfaction of consumers was observed in quartered and sliced cheese (in the case of cow’s milk cheeses) and sliced cheese from mixture of cow’s and sheep’s milk (also vacuum-packed). Overall, consumers showed less acceptance of cow’s milk pasta filata cheeses than CS cheeses. The addition of sheep’s milk reduced the amount of leachate from the vacuum-packed cheeses and did not cause considerable loss of gloss, as in the case of C cheeses. This is demonstrated, for example, by plasma/serum retained in the channels of the cheese structure. The leachate has proven to be an important criterion for food quality. 

Additionally, this study showed the potential usefulness of neural networks in dairy food processing. The developed predictive artificial neural network model allowed the estimation of the amount of water–fat serum released depending on the milk composition, cheese fragmentation, packing method, and storage time (within 24 h) after its unpacking, therefore, it can be useful in pasta filata cheese production process optimization.

## Figures and Tables

**Table 1:** Parametric data of fresh pasta filata cheese before and after portioning.

Parameters	Sphere	Quarters	Slices
Mass (g)	220	220	220
Volume (mL)	180	180	180
Total cheese area (cm^2^)	153.9	307.8	329.9
Surface of the outer layer (cm^2^)	153.9	153.9	153.9
Internal layer surface (cm^2^)	0	153.9	176.0
Total cheese area/Volume	0.86	1.71	1.83

**Table 2:** Gross composition and physicochemical characteristics of pasta filata cheeses from cow’s milk and a mixture with sheep’s milk.

Parameters	Pasta Filata Cheese		SEM
	C	CS	
Moisture (g/kg)	602.8 ^b^	568.8 ^a^	0.048
Fat (g/kg)	190.8 ^a^	188.8 ^a^	0.049
Protein (g/kg)	174.1 ^a^	204.0 ^b^	0.040
pH	5.15 ^a^	5.14 ^a^	0.000
Acidity (% lactic acid)	0.704 ^a^	0.707 ^a^	0.000
Hardness (g)	264.5 ^a^	259.9 ^a^	0.028
Stretching (mm)	127.9 ^a^	131.6 ^b^	0.054
Oiling-off (%)	3.17 ^a^	2.91 ^a^	0.001

^a,b^ Means within a row with different superscripts differ (*p* < 0.05). SEM: standard error of the mean (*n* = 6). C—from cow’s milk, CS—from cows and sheep milk in proportion 70:30.

**Table 3:** Sensory acceptability of pasta filata cheeses from cow’s milk and its mixture with sheep’s milk.

Samples			9	8	7	6	5	4	3	2	1	SK	*p*-Value	Dislike Responses (%)
C	B	W	48.8	29.8	20.2	1.2	0	0	0	0	0	1.48	0.002	0
		Q	47.6	21.4	22.6	6.0	2.4	0	0	0	0	1.62	0.005	0
		S	46.4	25.0	19.0	7.1	2.4	0	0	0	0	1.56	0.007	0
	V	W	67.9	25.0	4.8	2.4	0	0	0	0	0	2.42	<0.001	0
		Q	4.8	11.9	14.3	16.7	11.9	21.4	15.5	1.2	2.4	−0.25	0.515	40.5
		S	0	4.8	8.3	14.3	21.4	21.4	16.7	10.7	2.4	0.03	0.582	51.2
CS	B	W	85.7	9.5	3.6	1.2	0	0	0	0	0	2.93	<0.001	0
		Q	81.0	16.7	2.4	0.0	0	0	0	0	0	2.79	<0.001	0
		S	82.1	13.1	2.4	2.4	0	0	0	0	0	2.87	<0.001	0
	V	W	79.8	13.1	6.0	1.2	0	0	0	0	0	2.85	<0.001	0
		Q	78.6	15.5	4.8	1.2	0	0	0	0	0	2.80	<0.001	0
		S	6.0	8.3	9.5	17.9	21.4	22.6	10.7	2.4	1.2	0.37	0.398	34.9

SK: skewness; Milk (used in cheese production): C: Cow’s milk; CS: cow’s and sheep’s milk in proportion 70:30; Packaging (method): B: Packed with brine; V: Vacuum packed; Fragmentation (method): W: Whole sphere; Q: Quarters; S: Slices; 9—Like extremely, 8—Like very much, 7—Like moderately, 6—Like slightly, 5—Neither like nor dislike, 4—Dislike slightly, 3—Dislike moderately, 2—Dislike very much, 1—Dislike extremely.

**Table 4:** Consumer penalty analysis of the just-about-right (JAR) diagnostic attributes.

Samples			Shininess		Leachate		Compactness	
			Not Enough	Too Much	Not Enough	Too Much	Not Enough	Too Much
C	V	Q	61.3	–	–	88.2	82.4	–
		S	55.8	–	–	97.7	83.7	–
CS	V	S	32.4	–	–	80.6	71.0	12.9

(–) = indicates that less than 10% of consumers chose that JAR category; Milk (used in cheese production): C: Cow’s milk; CS: cow’s and sheep’s milk in proportion 70:30; Packaging (method): V: Vacuum packed; Fragmentation (method): Q: Quarters; S: Slices.

**Table 5:** Three-factorial analysis of variance for the amount of water–fat serum released from fresh pasta filata cheese made from cow’s milk and its mixture with sheep’s milk obtained within 24 h after unpacking.

Source of Variation	Sum of Square (SS)	*df*	Mean Square (MS)	*F*-Statistic	*p*-Value
Milk composition (MC)	668.65	1	668.65	2833.54	<0.0001
sample fragmentation (SF)	910.30	2	455.15	1928.78	<0.0001
packing method (PM)	489.37	1	489.37	2073.79	<0.0001
MC × SF	48.51	2	24.25	102.78	<0.0001
MC × PM	11.32	1	11.32	47.98	<0.0001
SF × PM	572.79	2	286.39	1213.65	<0.0001
MC × SF × PM	160.02	2	80.01	339.06	<0.0001
Error	5.66	24	0.24	-	-

**Table 6:** The basic information on the structure and training (learning) (El), test (Et) and validation (Ev) error values of MLP neural network adopted as the model water–fat serum release from fresh pasta filata cheese.

Architecture of Neural Network Model	Activation Function Hidden/Output Layer	Errors		
		E_l_	E_t_	E_v_
MLP 4(8)-11-1	Log/Lin	0.229	0.240	0.382

**Table 7:** Indicators used to evaluate the performance of the ANN-MLP model of water–fat serum release from pasta filata cheese.

Statistical Index	Data Set			
	Training	Test	Validation	Full
Number of observation points (N)	604	130	130	864
Coefficient of determination (R^2^)	0.997	0.996	0.996	0.997
Root mean square error (RMSE)	0.459	0.481	0.765	0.508

## Data Availability

The authors confirm that the data supporting the findings of this study are available within the article.
